# An Enhanced Moss Growth Optimization Algorithm with Outpost Mechanism and Early Stopping Strategy for Production Optimization in Tight Reservoirs

**DOI:** 10.3390/biomimetics10100704

**Published:** 2025-10-17

**Authors:** Chenglong Wang, Chengqian Tan, Youyou Cheng

**Affiliations:** 1 College of Petroleum Engineering, Xi’an Shiyou University, Xi’an 710065, China; 22111010001@stumail.xsyu.edu.cn; 2 Engineering Research Center of Development and Management for Low to Ultra-Low Permeability Oil & Gas Reservoirs in West China, Ministry of Education, Xi’an 710065, China; yycheng@xsyu.edu.cn; 3 School of Earth Sciences and Engineering, Xi’an Shiyou University, Xi’an 710065, China

**Keywords:** moss growth optimization, metaheuristics, early stopping strategy, outpost mechanism, tight reservoirs, bio-inspired optimization

## Abstract

Optimization algorithms play a crucial role in solving complex problems in reservoir geology and engineering, particularly those involving highly non-linear, multi-parameter, and high-dimensional systems. In the context of reservoir development, accurate optimization is essential for enhancing hydrocarbon recovery, improving production efficiency, and managing subsurface uncertainties. The Moss Growth Optimization (MGO) algorithm emulates the adaptive growth and reproductive strategies of moss. It provides a robust bio-inspired framework for global optimization. However, MGO often suffers from slow convergence and difficulty in escaping local optima in highly multimodal landscapes. To address these limitations, this paper proposes a novel algorithm called Strategic Moss Growth Optimization (SMGO). SMGO integrates two enhancements: an Outpost Mechanism (OM) and an Early Stopping Strategy (ESS). The OM improves exploitation by guiding individuals through multi-stage local search with Gaussian-distributed exploration around promising regions. This helps refine the search and prevents stagnation in sub-optimal areas. In parallel, the ESS periodically reinitializes the population using a run-and-reset procedure. This diversification allows the algorithm to escape local minima and maintain population diversity. Together, these strategies enable SMGO to accelerate convergence while ensuring solution quality. Its performance is rigorously evaluated on a suite of global optimization benchmarks and compared with state-of-the-art metaheuristics. The results show that SMGO achieves superior or highly competitive outcomes, with clear improvements in accuracy and stability. To demonstrate real-world applicability, SMGO is applied to production optimization in tight reservoirs. The algorithm identifies superior production strategies, leading to significant improvements in projected economic returns. This successful application highlights the robustness and practical value of SMGO. It offers a powerful and reliable optimization tool for complex engineering problems, particularly in strategic resource management for tight reservoir development.

## 1. Introduction

Optimization is central to solving non-linear and high-dimensional problems in engineering and science [1,2]. The core objective is to maximize desired outcomes—such as productivity, efficiency, or profit—or to minimize adverse effects, including cost, risk, or resource consumption [3]. Optimization is pervasive in diverse fields ranging from transportation and finance to drug design [4,5,6,7,8].

However, abstracting real-world scenarios into formal mathematical models inevitably exposes substantial complexities that challenge conventional solution methods. A major difficulty lies in the vast scale and dimensionality of the search space, as modern problems often involve thousands or even millions of decision variables, creating an immense landscape [9]. Non-linear relationships further complicate the analysis, as the effect of one variable depends intricately on others. Consequently, objective functions become rugged and multimodal, with numerous local optima that can trap simple search algorithms [10]. Additional difficulty arises from complex and conflicting constraints that restrict feasible solutions, intensifying the optimization challenge [11].

Classical mathematical programming and gradient-based methods, though powerful in convex and well-behaved problems, often fail in nonlinear, multimodal optimization tasks such as reservoir production optimization [12,13,14,15].

In response to the inherent limitations of traditional optimization methods, the scientific community has increasingly drawn inspiration from the natural world, giving rise to the field of biomimetics—the emulation of nature’s time-tested patterns and strategies to solve complex human problems. Within the computational realm, this paradigm has catalyzed the development of metaheuristic algorithms, which have emerged as powerful and versatile alternatives for tackling intractable optimization tasks [16,17]. Unlike their classical counterparts, metaheuristics are generally unbound by a problem’s mathematical characteristics, such as convexity or differentiability [18,19].

Despite their diverse origins, metaheuristic algorithms generally follow a common framework: they initialize a population of candidate solutions and iteratively refine it using specialized operators until a termination criterion is met. The design of these operators is crucial, as they must strategically balance two competing objectives: exploration, the ability to broadly sample the entire search space to discover new promising areas, and exploitation, the ability to intensively search within a known promising region to find the best possible solution [20,21]. Based on their primary source of inspiration, these algorithms are broadly classified into several major categories, most notably evolutionary, physics-based, and bio-behavioral algorithms [22].

Evolutionary algorithms are inspired by the principles of Darwinian natural selection and genetics. They simulate processes such as reproduction, mutation, recombination, and selection to evolve a population of solutions toward optimality. Prominent examples in this class include the Genetic Algorithm (GA) and Differential Evolution (DE) [23]. In contrast, physics-based algorithms draw analogies from physical laws and phenomena observed in the universe. Notable methods include Simulated Annealing (SA) [24], which mimics the cooling process of metals to reach a minimum energy state, and the Gravitational Search Algorithm (GSA) [25], which models the laws of gravity and mass interactions.

Finally, bio-behavioral algorithms emulate the collective or individual behaviors of living organisms. This category is dominated by Swarm Intelligence (SI) algorithms, which model the decentralized, self-organized problem-solving capabilities of social creatures. Well-known SI methods include Particle Swarm Optimization (PSO), inspired by the flocking of birds or schooling of fish [26], and Ant Colony Optimization (ACO) [27], which simulates the foraging behavior of ants using pheromone trails. The Moss Growth Optimization (MGO) algorithm [28], which forms the basis of this study, also falls squarely within this category.

Despite the proliferation and proven success of numerous metaheuristic algorithms, the quest for novel and more powerful optimization techniques remains an active and essential research direction. This ongoing quest is fundamentally justified by the “No Free Lunch” (NFL) theorem [29]. The NFL theorem formally posits that no single optimization algorithm can be universally superior to all others across the entire spectrum of possible optimization problems [30]. In essence, an algorithm that exhibits exceptional performance on one class of problems may perform poorly on another and vice versa.

This theorem illuminates an inherent trade-off in algorithm design: the specific strategies and operators that provide a competitive advantage for certain problem structures will inevitably become a liability when applied to others. Consequently, the NFL theorem underscores the critical necessity of continuously developing new algorithms or enhancing existing ones. For instance, while the MGO algorithm has shown promise in balancing exploration and exploitation through its unique bio-inspired mechanisms, it may exhibit limitations, such as slower convergence or a tendency to be trapped in local optima on particularly complex or high-dimensional problems. The NFL theorem, therefore, provides a strong impetus for tailoring and improving algorithms like MGO to better address the specific characteristics of challenging problem domains and to overcome identified weaknesses, thereby achieving superior practical performance where it matters most [28].

Tight reservoir production optimization is a prominent and highly challenging domain that demands advanced, tailored optimization algorithms [31]. In petroleum engineering, the effective management of unconventional resources like tight oil and gas reservoirs is critical for maximizing hydrocarbon recovery and ensuring the economic viability of energy projects [32]. This task involves interdependent decisions such as hydraulic fracture placement and design, well drilling and completion schedules, and dynamic control of production rates throughout the reservoir’s lifespan [33,34]. The primary objective is to maximize an economic indicator, usually Net Present Value (NPV), while satisfying geological and operational constraints.

Achieving this goal is exceptionally difficult. Tight reservoir systems are characterized by ultra-low permeability, significant geological heterogeneity, complex fracture network dynamics, and substantial subsurface uncertainty, all of which lead to highly non-linear and computationally expensive simulation models [35]. Consequently, traditional gradient-based methods are often ineffective, making metaheuristic algorithms a more suitable and powerful alternative for navigating this complex decision space [36].

Research in this domain has followed two main directions: enhancing computational efficiency and improving optimization algorithms. A major focus has been on reducing the heavy cost of high-fidelity reservoir simulations through fast proxy models or surrogates. For example, Chen et al. [37] developed a surrogate-assisted differential evolution algorithm to accelerate the optimization process. A parallel and equally critical research thrust concentrates on enhancing the internal mechanisms of the metaheuristic algorithms themselves to improve their search efficacy. Gao et al. [38] augmented the Drosophila Optimization Algorithm with multi-swarm and greedy selection mechanisms, leading to significantly better performance in oil production optimization.

Notably, recent work by Li et al. [39] enhanced the bio-inspired MGO algorithm by incorporating a Crisscross (CC) strategy and dynamic grouping. Their resulting CCMGO algorithm not only excelled on standard benchmarks but also achieved a superior NPV in a reservoir application, explicitly demonstrating that improving an algorithm’s internal information exchange and adaptive balancing can yield substantial gains. While extensive research has explored surrogate models and heuristic integrations, often relying on established optimizers like DE and PSO, these studies reveal a crucial insight: significant performance improvements are consistently realized by enhancing the core search behavior of the optimizer itself. The repeated success of such hybridization strategies underscores the pivotal role of the optimization engine’s architecture. This work advances the state of the art by enhancing the bio-inspired MGO algorithm through a novel combination of powerful strategies, rather than modifying the simulation framework.

The MGO algorithm, introduced by Zheng et al. [28], is a recently developed bio-behavioral metaheuristic inspired by the adaptive life cycle of moss. As detailed in the foundational paper, MGO translates the distinct reproductive strategies of moss into a computational framework for optimization. Its core mechanisms include a spore dispersal search for global exploration, mimicking how wind carries spores over long distances to colonize new areas, and a dual propagation search for local exploitation, which simulates the localized sexual and vegetative reproduction of moss to refine solutions in promising regions. This structured approach allows MGO to naturally balance wide-ranging exploration with intensive exploitation. While MGO has demonstrated considerable potential, it is not without limitations. On highly complex, multimodal landscapes, its convergence rate can be relatively slow, and like many metaheuristics, it can still be susceptible to premature convergence, becoming trapped in potent local optima and failing to discover the global best solution.

To address these shortcomings and enhance its overall performance, this paper introduces a novel and improved bio-inspired metaheuristic, termed the Strategic Moss Growth Optimization (SMGO) algorithm. The proposed SMGO method innovatively integrates the core MGO framework with two synergistic strategies: the Outpost Mechanism (OM) and the Early Stopping Strategy (ESS). The Outpost Mechanism is a multi-stage refinement process designed to bolster local search capabilities. It guides individuals to explore the vicinity of the current best solution in a structured yet random manner, with search steps approximated by a Gaussian distribution. This mechanism allows for a more precise and targeted exploitation of promising areas, directly addressing the risk of stagnation in sub-optimal states. Complementing this, the Early Stopping Strategy introduces a “run-and-reset” paradigm to counteract premature convergence. By periodically halting the optimization, recording the best-found solution, and reinitializing the population, ESS effectively injects diversity back into the search process. This forces the algorithm to explore entirely different regions of the solution space, significantly increasing its chances of escaping deep local minima. The synergistic integration of OM’s precise exploitation and ESS’s powerful diversification empowers SMGO to navigate complex optimization landscapes with greater efficiency and robustness.

The main contributions of this paper can be summarized as follows:

- We introduce the SMGO algorithm, an enhanced version of MGO that incorporates an Outpost Mechanism and an Early Stopping Strategy to improve search efficiency and solution quality.
- We provide a rigorous validation of SMGO’s performance on the CEC2017 benchmarks, demonstrating its superiority over numerous peer algorithms with statistical significance.
- We showcase SMGO’s real-world efficacy by applying it to a tight reservoir production optimization problem, where it successfully achieves a higher NPV.

The remainder of this paper is structured as follows: Section 2 reviews the original MGO. Section 3 presents the proposed SMGO algorithm. Section 4 contains the benchmark experiments and analysis. Section 5 details the engineering application. Finally, Section 6 offers our conclusions and outlines future work.

## 2. The Original MGO

The MGO algorithm, introduced by Zheng et al. in 2024 [29], is a bio-inspired mechanism. Since the general idea of MGO has been briefly introduced in the Introduction, here we only summarize its key operators and mathematical formulation relevant to the development of SMGO. By systematically modeling these biological behaviors, the primary mathematical framework of the MGO algorithm is structured as follows:

**1. Determination of wind direction:** MGO employs a novel “Determining Wind Direction” mechanism to guide the population’s evolution. This process leverages the spatial distribution of the majority of individuals relative to the current best solution to define an evolutionary path.

To begin, the population is partitioned dimension by dimension. For each j^th^ dimension. the value of the best individual, $M_{best}$, serves as a dividing threshold. Individuals with $X_{i,j}$ greater than or equal to this threshold are assigned to set $DX_{j1}$, while those with values less than it are placed in set $DX_{j2}$, The more populous of these two sets is then designated as $divX_{j}$, as specified in Equation (1).

(1) $$divX_{j}=\left\{\begin{array}{cc} DX_{j1}, & count\left(DX_{j1}\right)\geq count\left(DX_{j2}\right)\\ DX_{j2}, & count\left(DX_{j1}\right)<count\left(DX_{j2}\right) \end{array}\right.$$

where $count\left(\cdot \right)$ denotes a function that returns the cardinality of a set. This partitioning procedure is executed iteratively multiple times, and the final resulting set is defined as follows:

(2) $$di\nu X=\{M_{i}=\left(M_{i,1},M_{i,2},\ldots ,M_{i,dim}\right)|M_{i}\in \overset{dn}{\underset{j=1}{\cap }}\;di\nu X_{p_{j}},M_{i}\in X\}$$

where dn specifies the total number of partitioning iterations, and $p_{j}$ represents the randomly selected dimension used in this sequence. The process unfolds sequentially: the initial set is partitioned using the first selected dimension, the resulting subset is then partitioned using the second dimension, and so on, until all partitioning steps are completed to yield the final diνX set. Based on this resulting set, the wind direction vector is defined as follows:

(3) $$D_{-}wind=M_{best}-mean\left(divX\right)$$

**2. Spore dispersal search:** The Spore Dispersal Search serves as the primary exploration engine within the MGO algorithm, emulating the natural process by which moss spores are carried by wind to colonize new territories. This mechanism is responsible for generating new candidate solutions, enabling the algorithm to conduct a broad, global search across the solution space. It strategically models two distinct modes of dispersal corresponding to different wind conditions: a long-distance dispersal under stable winds for wide-ranging exploration and a short-distance dispersal under turbulent winds for more localized searching.

The generation of a new position $M_{i}^{new}$, from an existing individual $M_{i}$ is governed by the wind direction vector $D_{-}wind$ and a dynamically adjusted step size. A random number $r_{1}$ determines which of the two dispersal modes is activated. This is mathematically represented as:

(4) $$M_{i}^{new}=\{\begin{array}{cc} M_{i}+step1\cdot D_{-}wind & r_{1}>0.2\\ M_{i}+step2\cdot D_{-}wind & r_{1}\leq 0.2 \end{array}$$

The step sizes for these two modes are defined as follows:

(5) $$step1=w\cdot \left(r_{2}-0.5\right)\cdot E$$

(6) $$step2=0.1\cdot w\cdot \left(r_{3}-0.5\right)\cdot E\cdot \left[1+\frac{E}{2}\cdot \left(1+tanh\beta \sqrt{1-\beta^{2}}\right)\right]$$

where w is a constant (typically set to 2), $r_{2}$ and $r_{3}$ are random vectors in ([0, 1]), and E represents the wind strength, which decreases as the optimization progresses to transition from exploration to exploitation. E can be calculated as:

(7) $$E=1-\frac{FEs}{MaxFEs}$$

where FEs is the current number of function evaluations, and MaxFEs (maximum FEs) are allowed. The parameter β defined as the ratio of the size of divX to the total population size dynamically adjusts the step size based on the population’s convergence state.

In summary, the Spore Dispersal Search mechanism provides MGO with a powerful and adaptive exploration capability. By probabilistically switching between long- and short-range search steps and dynamically reducing the search radius over time via the wind strength factor E, it ensures a comprehensive exploration of the search space in the initial stages and a gradual transition toward finer-grained searching as the algorithm converges.

**3. Dual propagation search:** The Dual Propagation Search constitutes the primary exploitation strategy of the MGO algorithm, simulating the localized reproductive methods of moss—namely, sexual and asexual reproduction. This mechanism is designed to intensively search the promising regions identified around the current best solution, which is mathematically represented as:

(8) $$\left\{\begin{array}{cc} M_{i}^{new}=\left(1-act\right)\cdot M_{i}+act\cdot M_{best} & \;r_{4}>0.5\\ M_{i,j}^{new}=M_{best,j}+step3\cdot D\_wind_{j} & \;r_{4}\leq 0.5 \end{array}\right.$$

where act and step3 can be calculated as follows:

(9) $$act=\left\{\begin{array}{c} 1,\;\;\frac{1}{1.5-10\cdot r_{5}}\geq 0.5\\ 0,\;\;\frac{1}{1.5-10\cdot r_{5}}<0.5 \end{array}\right.$$

(10) $$step3=0.1\cdot \left(r_{6}-0.5\right)\cdot E$$

where $r_{4}$, $r_{5}$ and $r_{6}$ are random vectors in ([0, 1]).

In essence, the Dual Propagation Search provides MGO with a robust mechanism for local refinement. By probabilistically choosing between combining promising solutions and making small adjustments to the best-known solution, this strategy effectively balances the intensification of the search in elite regions, thereby accelerating convergence toward a high-quality optimum.

**4. Cryptobiosis mechanism**: The Cryptobiosis Mechanism is a distinctive feature of MGO designed to replace the conventional greedy selection process found in many metaheuristic algorithms. This mechanism draws inspiration from cryptobiosis in mosses—an extraordinary survival ability where the organism enters a state of metabolic dormancy to endure harsh conditions, only to revive later. In the context of optimization, this translates to an archive-based strategy that prevents the algorithm from prematurely discarding potentially valuable search positions.

Unlike a standard greedy approach, where a new solution immediately replaces its parent if it is better, the cryptobiosis mechanism in MGO utilizes an archive for each individual. Throughout the iterations, newly generated solutions are stored in this personal archive. The replacement of the current individual is not immediate but is triggered only when a specific condition is met, such as the archive reaching a predefined maximum size. When this trigger occurs, the algorithm evaluates all solutions stored in the archive and selects the best-performing one to replace the current individual. The archive is then cleared, and the process begins anew.

In summary, the MGO algorithm operates through a systematic, iterative process that integrates its distinct bio-inspired mechanisms. The algorithm commences by initializing a random population of moss individuals (candidate solutions). Then, within each iteration, the search process unfolds as follows: first, the Determination of Wind Direction mechanism is executed to establish a collective evolutionary trajectory.

**Figure 1:**
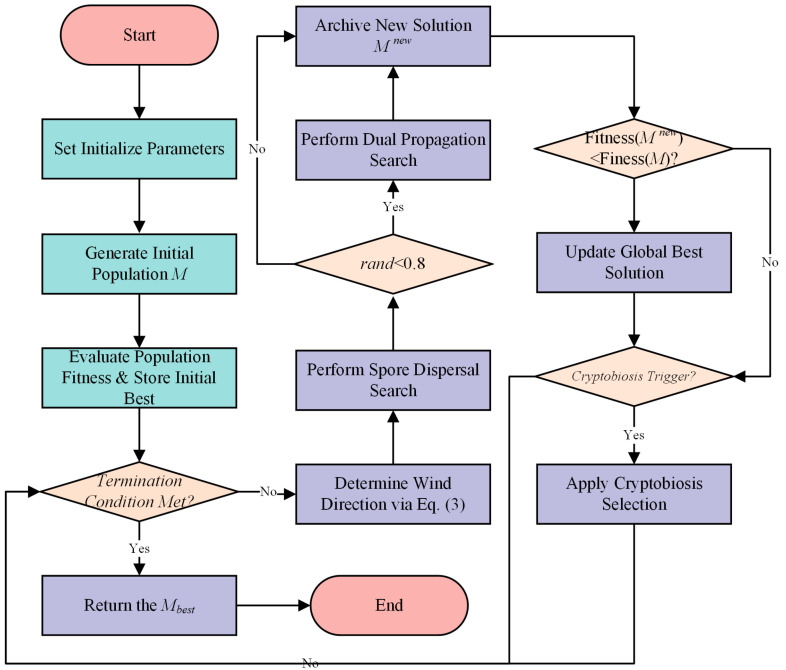
Flowchart of the MGO.

Following this, every individual in the population undergoes the Spore Dispersal Search phase, utilizing the calculated $D_{-}wind$ to generate a new candidate solution, thereby performing the primary exploration. Subsequently, with a high probability, the Dual Propagation Search is activated for each individual to conduct an intensive local search around the current best-found solution, facilitating exploitation. Finally, the newly generated solutions are managed by the Cryptobiosis Mechanism, which, instead of applying an immediate greedy selection, stores them in an archive. The current individuals are only updated with the best solution from their respective archives when a trigger condition is met. This entire iterative cycle of determining wind direction, exploration, exploitation, and archive-based selection is repeated until the maximum number of evaluations is reached. The best solution found throughout the entire process is then returned as the final output. The comprehensive flowchart of the MGO algorithm is illustrated in Figure 1.

## 3. Proposed SMGO

### 3.1. Outpost Mechanism

The OM is a sophisticated local search strategy designed to enhance the exploitation capabilities of MGO. It operates as a three-stage refinement process that guides individuals to meticulously explore the landscape around promising solutions, thereby preventing premature stagnation and improving the precision of the search.

**Stage 1: Initial Position Update**

In the first stage, a greedy selection is performed. Each individual, *X_i_*, is compared with its newly generated counterpart from the previous search phase, *X_new_*. The position with the superior fitness value is retained as the starting point for the subsequent exploration. This ensures that the search always proceeds from the most advantageous known location. The update rule can be expressed as:

(11) $$X_{i}^{\prime }=\mathrm{argmin}f\left(X_{i}\right),f\left(X_{new}\right)$$

where $X_{i}^{\prime }$ represents the updated position of the individual, and $f\left(\cdot \right),$ is the objective function.

**Stage 2: Gaussian-Distributed Exploration**

The second stage introduces a localized random search around the updated position $X_{i}^{\prime }$, To simulate a focused yet comprehensive exploration of the immediate neighborhood, this search is guided by a standard normal (Gaussian) distribution. A perturbation vector $\vec{\delta }$, is generated, where each component is drawn from a Gaussian distribution with a mean μ of 0 and a standard deviation σ of 1. This vector is then scaled and added to the individual’s position to create a new trial solution, $V_{i}$:

(12) $$V_{i}=X_{i}^{\prime }+\alpha \cdot \vec{\delta }\;\;\;\;\;\;\;\;\;\;\;\;\;\;\;\;\;\;\;\delta_{d}∼N\left(0,1\right)$$

where $V_{i}$ is the trial vector for individual i*,* $\vec{\delta }$ is the Gaussian perturbation vector, $\delta_{d}$ is its component in dimension d, and α is a scaling factor that controls the search radius, which can be dynamically adjusted during the optimization process.

**Stage 3: Adaptive Tendency Adjustment**

In the final stage, an adaptive adjustment is made based on the quality of the trial solution, $V_{i}$. The algorithm evaluates whether the exploration has led to an improvement. If the fitness of $V_{i}$, is better than that of $X_{i}^{\prime }$, the search is considered successful, and the final updated position $X_{i}^{final}$ is biased further in the direction of the successful move $\left(V_{i}-X_{i}^{\prime }\right)$, Conversely, if no improvement is found, the final position is adjusted away from the unsuccessful direction to encourage exploration elsewhere. This tendency-based update is formulated as:

(13) $$X_{i}^{final}=X_{i}^{\prime }+\mathrm{sign}\left(f\left(X_{i}^{\prime }\right)-f\left(V_{i}\right)\right)\cdot \beta \cdot \left(V_{i}-X_{i}^{\prime }\right)$$

where $\mathrm{sign}\left(\cdot \right)$ is the sign function, which returns +1 for a positive argument (indicating improvement) and −1 otherwise. The parameter β is the learning rate that controls the magnitude of the final adjustment, which is set to 0.5. Through this three-stage process, the Outpost Mechanism provides a robust and adaptive local search that significantly enhances MGO’s ability to converge on high-quality solutions.

### 3.2. Early Stopping Strategy

While the core mechanisms of the MGO algorithm provide a robust framework for optimization, its performance can be susceptible to two common challenges inherent in metaheuristic search. Firstly, MGO’s convergence can sometimes be gradual, and in later stages of the optimization, the population may stagnate around a potent local optimum, leading to inefficient use of computational resources as further iterations yield only marginal improvements. Secondly, for highly multimodal or deceptive problem landscapes, a single, continuous run—even with MGO’s built-in diversity mechanisms—may still commit to a suboptimal region of the search space early on and fail to discover the true global optimum.

To counteract these potential issues, we integrate an ESS into the MGO framework. The ESS is not a continuous search operator but rather a structural modification to the overall optimization process. It divides the total allotted number of function evaluations into multiple independent search phases. The strategy is implemented as follows:

1. Partial Execution and Archiving: The MGO algorithm is executed for a predetermined portion of the total computational budget (e.g., two-thirds of the total function evaluations). Upon reaching this intermediate stopping point, the best solution found during this first phase is recorded and archived.
2. Population Reinitialization: After the first phase concludes, the entire population of moss individuals is discarded. A completely new, randomly initialized population is then generated across the entire search space. This step effectively erases any “memory” of the previous search, including any biases toward the previously explored suboptimal region.
3. Resumed Execution: The MGO algorithm is then run for the remainder of the computational budget, starting fresh with the newly initialized population. This second phase allows the algorithm to explore entirely different regions of the solution landscape, providing a new opportunity to locate the global optimum.
4. Final Greedy Selection: Once all phases are complete, the best solution archived from the first phase is compared with the best solution found during the second phase. The superior of the two is then selected as the final output of the entire optimization process.

By partitioning the search into distinct, memory-less phases, the Early Stopping Strategy provides a powerful mechanism for escaping deep-rooted local optima. This “reset” functionality fundamentally enhances the algorithm’s global search capability without increasing the total computational cost. It acts as a safeguard against premature convergence, significantly improving both the reliability and the final solution quality of the MGO algorithm, particularly on challenging, high-dimensional optimization problems.

### 3.3. The Proposed SMGO

This section formally introduces the proposed SMGO algorithm. The SMGO framework enhances the standard MGO by strategically integrating the OM and structuring the overall search process with an ESS.

**Figure 2:**
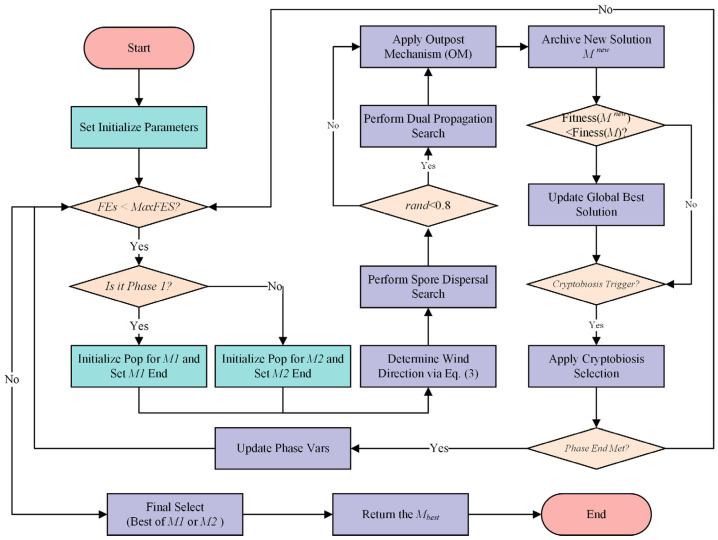
Flowchart of the SMGO.

After the initial population is generated, the optimization process, governed by the ESS, is divided into distinct search phases. Within each phase, the iterative loop of the algorithm begins by applying the standard MGO update rules, including the determination of wind direction, spore dispersal, and dual propagation searches. Subsequently, the OM is employed on the resulting population as a local refinement step to enhance exploitation and generate new, high-precision trial solutions. The cryptobiosis mechanism then manages the selection of individuals for the next generation. This iterative process repeats until the function evaluation budget for the current phase is exhausted. Upon completion of all phases, the best solutions from each phase are compared, and the global best is returned. The complete workflow of the SMGO algorithm is depicted in the flowchart in Figure 2.

Algorithm 1 provides the pseudo-code for the SMGO.
**Algorithm 1.** Pseudo-code of the SMGOSet parameters: MaxFES (Total evaluations), N (Population size), dim (Dimension), R (Archive size), ESS_Split_Point GlobalBest.Cost ← ∞ FEs ← 0 Run_Phase ← 1 **While**
FEs < MaxFES
   **//Phase 1: Initial Run**  **IF** Run_Phase = 1
    Population ← InitializePopulation(N, D)     FEs ← FEs + N    $Best\_Phase1\;\leftarrow \;GetBestSolution\left(Population\right)$    Current_Phase_End ← ESS_Split_Point  **End IF**  **//Phase 2: Reinitialization after Early Stop**  **IF** Run_Phase = 2    $Population\;\leftarrow \;InitializePopulation\left(N,\;D\right)$    FEs ← FEs + N    Current_Phase_End ← MaxFES    Run_Phase ← 3//Mark phase 2 as started to prevent re-entry  **End IF**  **While** FEs < Current_Phase_End    **//Standard MGO Main Loop**    **For** i = 1 to N
      **//MGO Step 1**      $D\_wind\;\leftarrow \;Determine\_Wind\_Direction\left(Population,\;GlobalBest\right)$
      **//Spore Dispersal Search (Exploration)—MGO Step 2**      $New\_Sol\_Explore\;\leftarrow \;Spore\_Dispersal\_Search\left(Population\left(i\right),\;D\_wind\right)$
      **//Dual Propagation Search (Exploitation)—MGO Step 3**      **IF** rand() < 0.8        $New\_Sol\_Exploit\;\leftarrow \;Dual\_Propagation\_Search\left(New\_Sol\_Explore,\;GlobalBest,\;D\_wind\right)$      **Else**        New_Sol_Exploit ← New_Sol_Explore      **End IF**      FEs ← FEs + 1      **//Outpost Mechanism (Local Refinement)**      $Final\_Sol\;\leftarrow \;Outpost\_Mechanism\left(New\_Sol\_Exploit\right)$      FEs ← FEs + 1//Account for OM evaluation      **//Cryptobiosis Mechanism—MGO Step 4**      $Population\left(i\right)\;\leftarrow \;Cryptobiosis\_Update\left(Population\left(i\right),\;Final\_Sol,\;R\right)$    **End For**    **//Update Global Best for the current phase**    $CurrentBest\;\leftarrow \;GetBestSolution\left(Population\right)$    **IF** Run_Phase = 1 **And** CurrentBest.Cost < Best_Phase1.Cost      Best_Phase1 ← CurrentBest    **End IF**    **IF** GlobalBest.Cost > CurrentBest.Cost      GlobalBest ← CurrentBest    **End IF**  **End While**   **//Trigger for the next phase (Early Stopping)**  **IF** Run_Phase = 1    Run_Phase ← 2  **End IF**  **End While** **IF**
Best_Phase1.Cost < GlobalBest.Cost GlobalBest ← Best_Phase1 **End IF** **Return**
GlobalBest

The computational complexity of the proposed SMGO algorithm arises from four main components: population initialization, the standard MGO search operators, the integrated OM, and the cryptobiosis mechanism. Let T denote the maximum number of iterations, N the population size, D the problem dimension, and R the maximum archive size for the cryptobiosis mechanism. The initialization phase incurs a one-time cost of O(N × D). During each of the T iterations, the dominant operations include the MGO search operators with complexity O(N × D), the Outpost Mechanism with an additional O(N × D), and the cryptobiosis mechanism with O(N × R) for archive management. Consequently, the overall complexity can be expressed as O(N × D) + T × (O(N × D) + O(N × R)). Simplifying by the dominant terms yields O(T × N×(D + R)), which for most practical cases—where the problem dimension D is much larger than the archive size R—further reduces to O(T × N×D).

## 4. Experimental Results and Analysis

To rigorously validate the performance of the proposed SMGO algorithm, a comprehensive series of numerical experiments was performed. The evaluation was primarily conducted on a challenging set of global optimization problems to thoroughly assess SMGO’s search efficacy and robustness. For a fair and direct comparison against other state-of-the-art methods, all experiments were executed under standardized conditions.

The population size for all algorithms was set to 30, and the problem dimensionality was fixed at 30. Each optimization run was allocated a maximum computational budget of 300,000 function evaluations. To account for the stochastic nature of metaheuristic algorithms, every experiment was independently repeated 30 times. The final performance was then quantified using statistical metrics, primarily the mean and standard deviation of the results obtained across these trials, ensuring a reliable and unbiased assessment.

### 4.1. Benchmark Functions Overview

The performance of the proposed SMGO algorithm was evaluated on the 29 benchmark functions of the CEC2017 test suite. The CEC2017 suite is among the most widely adopted benchmark sets in the evolutionary computation community, providing a standardized and rigorous basis for evaluating global optimization algorithms. This widely used suite, detailed in [40], provides a diverse set of challenges by including unimodal, simple multimodal, hybrid, and composition functions. This variety ensures a comprehensive assessment of an algorithm’s exploitation, exploration, and ability to handle complex fitness landscapes.

### 4.2. Comparative Analysis on Benchmark Functions

This section presents a comparative performance evaluation of the proposed SMGO against the original MGO and nine other well-established metaheuristic algorithms on the 29 benchmark functions from the CEC2017 suite. The selected peer algorithms include the Whale Optimization Algorithm (WOA) [41], Grey Wolf Optimizer (GWO) [42], Moth-Flame Optimization (MFO) [43], Hunger Games Search (HGS) [44], Sine Cosine Algorithm (SCA) [45], Slime Mould Algorithm (SMA) [46], GSA [25], a high-performance optimizer referenced as Colony Predation Algorithm (CPA) [47], and the Water Flow Optimization (WFO) [48]. To ensure a fair comparison, all algorithms were implemented with their control parameters configured based on common recommendations in their original publications.

Table 1 provides a comprehensive comparison of the twelve algorithms on the CEC2017 benchmark suite. For each algorithm and function, the table reports the average fitness (Avg) and standard deviation (Std) obtained over 30 independent runs. To facilitate an overall performance assessment, the table also includes the final rank of each algorithm as determined by the Friedman test, alongside a pairwise statistical summary (+/=/−) indicating the performance of MGO relative to your new algorithm (SMGO).

The results presented in Table 1 unequivocally demonstrate that SMGO achieves the best overall performance, attaining the lowest average Friedman rank of 1.8966. This places it significantly ahead of the second-best performer, WFO, which secured a rank of 2.4483. The original MGO ranked fourth with an average rank of 4.1034. The pairwise comparison reveals that SMGO significantly outperformed its predecessor, MGO, on 19 functions, was statistically equivalent on 7 functions, and was outperformed by MGO on only 3 functions. This indicates a substantial performance improvement across the vast majority of the test cases. Other strong performers included CPA and GSA, with average ranks of 4.069 and 4.8621, respectively. In contrast, algorithms such as WOA, MFO, and SCA ranked lower, highlighting SMGO’s superior efficacy in navigating these complex optimization landscapes. Furthermore, the standard deviation values for SMGO were consistently competitive, and on several functions (e.g., F1, F5, F6), were among the lowest, suggesting robust and stable performance.

Table 2 further substantiates the superiority of SMGO by presenting the *p*-values from the Wilcoxon signed-rank test. This test was conducted to compare SMGO against each of the other eleven algorithms on the CEC2017 benchmark suite, using a significance level of *p* = 0.05. The results reveal that SMGO achieves statistically significant improvements (*p* < 0.05) over the majority of competitor algorithms—including WOA, GWO, MFO, HGS, SCA, SMA, and GSA—on nearly all 29 functions, as indicated by the overwhelmingly small *p*-values.

In the critical comparison against its direct predecessor, MGO, SMGO demonstrates a statistically significant advantage on numerous challenging functions, such as F2, F9, F10–F14, F17, F18, F21, F27–F29. This confirms that the architectural modifications are not just beneficial on average but provide a decisive edge on specific, difficult problems. Against the other top-tier performers, WFO and CPA, SMGO also exhibits a compelling performance profile. For instance, it secures statistically significant victories over WFO on F1, F2, F7, F8, and F20, while remaining highly competitive on others. These statistical findings confirm that the integration of the Outpost Mechanism and the Early Stopping Strategy leads to tangible and verifiable enhancements in SMGO’s optimization capabilities.

**Figure 3:**
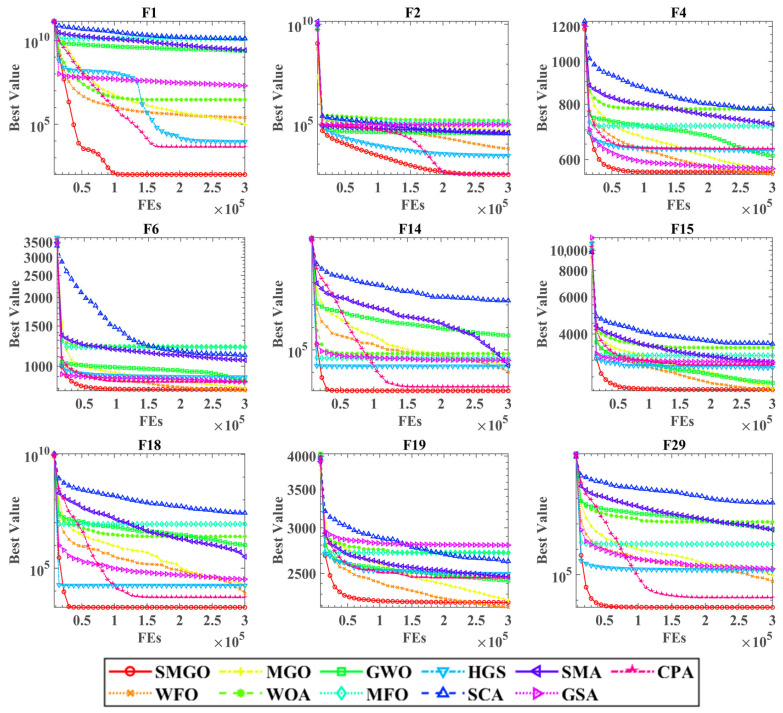
Convergence curves of the SMGO on benchmarks with other algorithms.

Figure 3 displays the convergence curves of all twelve algorithms on nine representative benchmark functions (F1, F2, F4, F6, F14, F15, F18, F19, and F29), with SMGO highlighted by a bold red line with circles. The horizontal axis represents function evaluations (FEs) up to 300,000 and the vertical axis shows the best fitness value achieved; a logarithmic scale is used for the *y*-axis on F1, F2, F14, F18 and F29 to better visualize performance on these problems.

The convergence plots visually demonstrate SMGO’s superior performance characteristics. On most functions, especially the challenging multimodal and hybrid ones like F1, F2, F14, F18, and F29, SMGO converges dramatically faster and to significantly better solutions than all competitors. This is evidenced by its steep initial fitness decline, confirming a highly effective exploration phase, likely amplified by the Early Stopping Strategy, followed by a sustained and deep convergence, showcasing the precision of the Outpost Mechanism. Even on functions where other algorithms perform well, such as F4 and F6, SMGO’s curve consistently lies at the bottom, indicating it finds the highest quality solutions. In nearly all depicted cases, SMGO’s curve lies substantially below that of the original MGO, with the performance gap being particularly pronounced on F1, F14, F18, and F29. These results visually confirm that the proposed strategies significantly enhance both the convergence speed and final solution quality of the algorithm.

Moreover, additional experiments on several simple benchmark functions, as shown in Table 3, confirm the generality of SMGO. The algorithm maintains excellent performance on both simple and complex problems, achieving near-zero errors with minimal variance on basic functions such as Sphere, Schwefel, and Rosenbrock. These results demonstrate that SMGO is robust, stable, and well-suited for optimization tasks of varying complexity.

In summary, the comprehensive analysis of the benchmarks confirms the outstanding performance of the proposed SMGO algorithm. Through superior Friedman ranking, statistically significant improvements validated by the Wilcoxon test, and consistently faster and deeper convergence curves, SMGO has demonstrated high effectiveness and robustness, primarily owing to the complementary roles of its two mechanisms. The OM enhances information exchange among individuals, thereby reducing the likelihood of premature convergence to local optima. Meanwhile, the ESS improves the balance between exploration and exploitation by terminating redundant iterations once convergence has plateaued.

## 5. Application to Tight Reservoirs Production Optimization

The primary goal of tight reservoir production optimization is to design operational strategies for production and injection wells that maximize the project’s economic return, typically measured by net present value (NPV). This task is especially challenging in tight reservoirs, where managing complex hydraulic fracture networks and forecasting long-term production under substantial geological uncertainty create an immense and computationally expensive search space. The high dimensionality and strong non-linearity between well controls and reservoir performance further limit the effectiveness of traditional gradient-based methods, making metaheuristic algorithms particularly well-suited for identifying robust, near-optimal solutions.

In this study, the proposed SMGO algorithm is applied to a benchmark tight reservoir case study, implemented using the MATLAB Reservoir Simulation Toolbox 2024b (MRST). Its performance is evaluated by comparing the final NPV achieved against that of several other leading optimizers. The optimization target is the maximization of the NPV, which serves as the objective function for the optimization problem, defined in Equation (15):

(14) $$NPV\left(x,z\right)=\overset{n}{\underset{t=1}{\sum }}\Delta t\cdot \frac{\left(Q_{o,t}\cdot r_{o}\right)-\left(Q_{w,t}\cdot r_{w}\right)-\left(Q_{i,t}\cdot r_{i}\right)}{{\left(1+b\right)}^{p_{t}}}$$

where x is the vector of control variables, n is the number of time steps, Δt is the duration of each step. At each time step t, $Q_{o,t}$, $Q_{w,t}$, and $Q_{i,t}$ are the oil production, water production, and water injection rates, with corresponding economic parameters $r_{o}$, (oil price), $r_{w}$, (water disposal cost) and $r_{i}$ (water injection cost). The term b represents the annual discount rate applied over the time $p_{t}$ in years.

### 5.1. Reservoir Model Description

**Figure 4:**
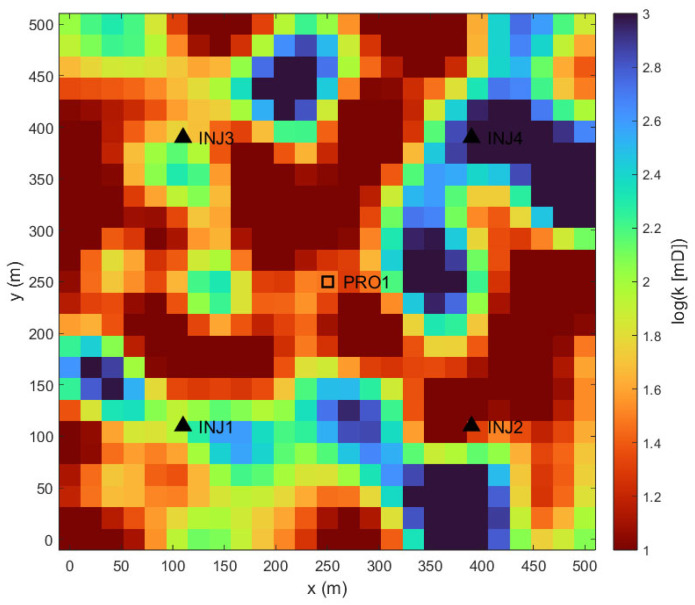
Distribution of Log-Permeability.

This study’s practical application is demonstrated on a two-dimensional, synthetic reservoir model designed to emulate the complex geological conditions of a tight sandstone formation developed with hydraulic fracturing. As shown in Figure 4, the reservoir features a central fractured producer (PRO1) surrounded by four injectors (INJ1–INJ4) in an inverted five-spot configuration. The domain is represented by a 25 × 25 Cartesian grid (625 cells), where each cell is 20 m × 20 m. A constant porosity of 0.12 is used throughout, reflecting typical tight reservoir characteristics.

The defining feature of this model is its significant permeability heterogeneity, a critical aspect of tight reservoirs where production is governed by the stimulated fracture network. Figure 4 illustrates the spatial distribution of the base-10 logarithm of permeability, log(k). The high-permeability channels (warm colors) represent the stimulated reservoir volume (SRV), which act as primary fluid conduits, while the surrounding low-permeability matrix (cool colors) forms flow baffles. This intricate structure creates tortuous flow paths, making efficient waterflood management a complex optimization task. The reservoir is initialized with oil and connate water.

The optimization objective is to maximize the NPV over a 1500-day production lifetime, which is discretized into 15 control periods of 100 days each. The decision variables are the operational controls for the five wells across these 15 periods, resulting in a 75-dimensional optimization problem. The NPV calculation, serving as the algorithm’s fitness function, is based on an oil price of 80 USD/STB, water injection and processing costs of 5 USD/STB each, and an annual discount rate of 10% to account for the time value of capital.

### 5.2. Analysis and Discussion of Experimental Results

This section presents a detailed performance analysis of the proposed SMGO, the original MGO, and nine other metaheuristics on the tight reservoir production optimization problem. To ensure a robust comparison for this computationally intensive application, each algorithm was executed multiple times independently. The key statistical metrics, including the mean, standard deviation (Std), best, and worst NPV achieved, are reported in Table 4.

The results summarized in Table 4 clearly demonstrate the superior performance of SMGO. It achieved the highest mean NPV of 1.0528 × 10^8^ USD, underscoring its enhanced capability to consistently identify high-value production strategies. Furthermore, SMGO exhibited the lowest standard deviation of 1.1534 × 10^6^ USD, indicating exceptional stability and reliability in its outcomes compared to all its peers, including the next best performers, WFO (1.8950 × 10^6^) and CPA (2.0547 × 10^6^).

This performance gain is particularly notable when compared to the original MGO, which not only achieved a significantly lower mean NPV of 9.7145 × 10^7^ USD but also showed nearly double the performance variance with a standard deviation of 2.1881 × 10^6^. This demonstrates that the synergistic combination of the Outpost Mechanism and the Early Stopping Strategy enhances both the global search capability and the local convergence precision of the algorithm. These numerical results highlight SMGO’s dual strengths: it effectively navigates the complex, high-dimensional search space of tight reservoir management to maximize economic returns while maintaining remarkable consistency across multiple independent runs.

**Figure 5:**
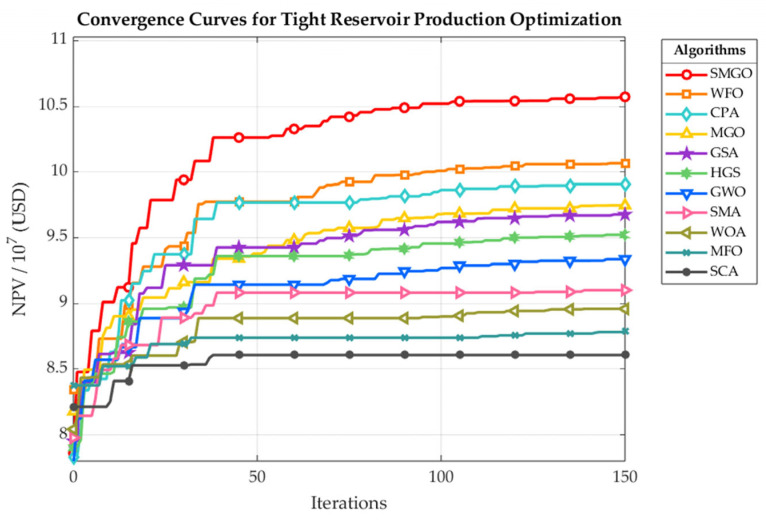
NPV Convergence Trajectories for the Tight Reservoir Problem.

Figure 5 provides a visual comparison of the convergence dynamics for SMGO and the ten other algorithms on the tight reservoir optimization problem. The horizontal axis represents the number of iterations, while the vertical axis shows the best-so-far NPV. The plot clearly illustrates that SMGO not only converges to the highest final NPV but also demonstrates the most rapid and consistent rate of improvement among all tested methods.

SMGO, represented by the solid red line, exhibits a remarkably aggressive search trajectory within the initial 40 iterations, quickly surpassing all competitors and establishing a commanding lead that it never relinquishes. In contrast, the second and third-best algorithms, WFO and CPA, also show strong initial performance but begin to stagnate much earlier and stabilize at a visibly lower economic return. The performance gap between SMGO and its predecessor, MGO, is particularly significant; MGO displays a much slower rate of improvement and converges to a substantially lower NPV, which powerfully underscores the efficacy of the integrated Outpost Mechanism and Early Stopping Strategy. The remaining algorithms are stratified in distinct performance tiers, with methods like GSA and HGS showing moderate success, while others such as MFO and SCA lag considerably, finding solutions of significantly lower economic value.

SMGO is particularly effective for tight reservoir production optimization. Specifically:

- The Outpost Mechanism enhances local refinement, which is especially beneficial for the highly non-linear response surfaces of reservoir simulations.
- The Early Stopping Strategy avoids unnecessary evaluations during stagnation, which is crucial given the high computational cost of reservoir simulations.
- Together, these mechanisms achieve a balanced trade-off between exploration and exploitation, enabling SMGO to deliver high-quality solutions under strict computational budgets.

These features make SMGO especially efficient in this domain, even though, consistent with the no free lunch theorem, it may not always dominate on unrelated problem classes.

## 6. Conclusions

This work introduced the SMGO algorithm, a targeted enhancement of the original MGO aimed at addressing its known limitations in convergence speed and its susceptibility to local optima. This was accomplished through the novel integration of two complementary mechanisms: the OM, which intensifies local search precision, and the ESS, which reinvigorates the global search to prevent premature stagnation. The resulting framework is designed to preserve population diversity while significantly accelerating progress toward high-quality solutions, thus improving the algorithm’s overall optimization efficacy.

The proposed SMGO was validated through empirical testing. On the CEC2017 test suite, it achieved top performance, ranking first in Friedman tests and showing a significant advantage over MGO and other state-of-the-art algorithms. In a high-fidelity tight reservoir optimization case, SMGO consistently produced the highest mean NPV, confirming its effectiveness and reliability for complex engineering applications.

Future work will pursue adaptive parameter control for OM and ESS, extend SMGO to multi-objective problems, and apply it to petroleum engineering tasks such as well placement optimization and automatic history matching. Expanding the test portfolio will further validate its versatility.

## Figures and Tables

**Table 1:** Results of the SMGO and Other Algorithms on CEC2017.

	F1		F2		F3	
	Avg	Std	Avg	Std	Avg	Std
SMGO	1.0000 × 10^2^	3.8873× 10^−14^	3.0080 × 10^2^	1.6811 × 10^0^	4.2981 × 10^2^	3.0367 × 10^1^
MGO	9.6898 × 10^4^	7.6967 × 10^4^	4.8814 × 10^4^	9.6014 × 10^3^	4.9480 × 10^2^	1.5828 × 10^1^
WOA	2.9332 × 10^6^	2.1041 × 10^6^	1.5200 × 10^5^	6.4283 × 10^4^	5.4437 × 10^2^	4.2191 × 10^1^
GWO	2.3823 × 10^9^	1.7326 × 10^9^	3.3121 × 10^4^	8.8382 × 10^3^	5.7320 × 10^2^	6.8269 × 10^1^
MFO	1.3109 × 10^10^	7.7727 × 10^9^	1.1197 × 10^5^	6.0916 × 10^4^	1.5093 × 10^3^	1.1889 × 10^3^
HGS	8.9829 × 10^3^	8.2208 × 10^3^	2.6204 × 10^3^	5.0153 × 10^3^	4.9502 × 10^2^	2.7678 × 10^1^
SCA	1.2916 × 10^10^	2.0357 × 10^9^	3.4792 × 10^4^	7.3002 × 10^3^	1.4017 × 10^3^	1.9186 × 10^2^
SMA	2.6404 × 10^9^	1.2014 × 10^9^	3.8142 × 10^4^	7.9024 × 10^3^	6.1394 × 10^2^	7.7361 × 10^1^
GSA	1.9707 × 10^7^	2.4690 × 10^6^	9.4514 × 10^4^	1.2566 × 10^4^	4.7403 × 10^2^	2.3330 × 10^1^
CPA	4.2024 × 10^3^	4.8625 × 10^3^	3.0000 × 10^2^	1.3965 × 10^−7^	4.7355 × 10^2^	3.0154 × 10^1^
WFO	2.4949 × 10^5^	7.3635 × 10^5^	5.9201 × 10^3^	1.9930 × 10^3^	4.8943 × 10^2^	1.1960 × 10^1^
	**F4**		**F5**		**F6**	
	**Avg**	**Std**	**Avg**	**Std**	**Avg**	**Std**
SMGO	5.6226 × 10^2^	1.5023 × 10^1^	6.0000 × 10^2^	4.5861 × 10^−4^	7.9101 × 10^2^	1.6666 × 10^1^
MGO	5.6600 × 10^2^	9.8476 × 10^0^	6.0000 × 10^2^	1.1640 × 10^−4^	8.0139 × 10^2^	1.4899 × 10^1^
WOA	7.8058 × 10^2^	6.2404 × 10^1^	6.6595 × 10^2^	1.0182 × 10^1^	1.2211 × 10^3^	9.6164 × 10^1^
GWO	6.1196 × 10^2^	3.6418 × 10^1^	6.0715 × 10^2^	3.1805 × 10^0^	8.6316 × 10^2^	4.4216 × 10^1^
MFO	7.1483 × 10^2^	4.9747 × 10^1^	6.4254 × 10^2^	1.1981 × 10^1^	1.2127 × 10^3^	2.7160 × 10^2^
HGS	6.2920 × 10^2^	3.0635 × 10^1^	6.0128 × 10^2^	1.1065 × 10^0^	8.9494 × 10^2^	4.4822 × 10^1^
SCA	7.7976 × 10^2^	1.8954 × 10^1^	6.4985 × 10^2^	6.4595 × 10^0^	1.1200 × 10^3^	3.7616 × 10^1^
SMA	7.2161 × 10^2^	4.0761 × 10^1^	6.4337 × 10^2^	6.7870 × 10^0^	1.0628 × 10^3^	4.7570 × 10^1^
GSA	5.7207 × 10^2^	1.1162 × 10^1^	6.0185 × 10^2^	1.0086 × 10^0^	8.5390 × 10^2^	1.0042 × 10^1^
CPA	6.3323 × 10^2^	3.0619 × 10^1^	6.0000 × 10^2^	1.8101 × 10^−4^	8.4980 × 10^2^	2.9498 × 10^1^
WFO	5.5476 × 10^2^	1.0299 × 10^1^	6.0000 × 10^2^	3.8313 × 10^−5^	7.7956 × 10^2^	8.1769 × 10^0^
	**F7**		**F8**		**F9**	
	**Avg**	**Std**	**Avg**	**Std**	**Avg**	**Std**
SMGO	8.5937 × 10^2^	1.1533 × 10^1^	9.9822 × 10^2^	9.0665 × 10^1^	3.0485 × 10^3^	3.1445 × 10^2^
MGO	8.6783 × 10^2^	1.2760 × 10^1^	9.4740 × 10^2^	5.3726 × 10^1^	4.5059 × 10^3^	4.4595 × 10^2^
WOA	9.9754 × 10^2^	4.7410 × 10^1^	8.5245 × 10^3^	2.9149 × 10^3^	6.2197 × 10^3^	7.8647 × 10^2^
GWO	8.8692 × 10^2^	2.2230 × 10^1^	1.7444 × 10^3^	4.7345 × 10^2^	3.8703 × 10^3^	6.0714 × 10^2^
MFO	9.9457 × 10^2^	4.0300 × 10^1^	7.7235 × 10^3^	2.2964 × 10^3^	5.3633 × 10^3^	7.6880 × 10^2^
HGS	9.1735 × 10^2^	3.2538 × 10^1^	3.8929 × 10^3^	1.0893 × 10^3^	3.9916 × 10^3^	5.6463 × 10^2^
SCA	1.0435 × 10^3^	1.6618 × 10^1^	5.4466 × 10^3^	9.4177 × 10^2^	8.1280 × 10^3^	2.3845 × 10^2^
SMA	9.6816 × 10^2^	2.3318 × 10^1^	5.4868 × 10^3^	1.1035 × 10^3^	5.8393 × 10^3^	5.6292 × 10^2^
GSA	8.6385 × 10^2^	9.4510 × 10^0^	9.0620 × 10^2^	8.5850 × 10^−1^	3.3251 × 10^3^	3.2635 × 10^2^
CPA	9.1618 × 10^2^	1.6313 × 10^1^	2.3654 × 10^3^	7.0149 × 10^2^	4.0917 × 10^3^	4.1614 × 10^2^
WFO	8.5166 × 10^2^	6.4020 × 10^0^	9.0279 × 10^2^	3.2228 × 10^0^	3.8067 × 10^3^	3.6143 × 10^2^
	**F10**		**F11**		**F12**	
	**Avg**	**Std**	**Avg**	**Std**	**Avg**	**Std**
SMGO	1.1245 × 10^3^	1.0734 × 10^1^	5.4718 × 10^3^	4.8070 × 10^3^	1.3429 × 10^3^	1.8099 × 10^1^
MGO	1.1910 × 10^3^	2.7732 × 10^1^	1.0449 × 10^6^	7.2890 × 10^5^	3.1422 × 10^4^	2.3693 × 10^4^
WOA	1.5521 × 10^3^	2.3917 × 10^2^	3.9568 × 10^7^	3.0000 × 10^7^	1.6583 × 10^5^	1.0255 × 10^5^
GWO	1.7179 × 10^3^	6.6656 × 10^2^	5.8792 × 10^7^	8.3457 × 10^7^	7.8173 × 10^5^	3.6110 × 10^6^
MFO	3.2606 × 10^3^	2.4052 × 10^3^	3.5410 × 10^8^	8.4543 × 10^8^	7.1824 × 10^7^	2.6846 × 10^8^
HGS	1.2110 × 10^3^	4.4671 × 10^1^	9.1587 × 10^5^	6.7726 × 10^5^	2.8640 × 10^4^	2.3366 × 10^4^
SCA	2.0731 × 10^3^	2.3424 × 10^2^	1.3121 × 10^9^	3.2431 × 10^8^	3.8825 × 10^8^	1.4446 × 10^8^
SMA	1.5241 × 10^3^	1.0495 × 10^2^	1.1533 × 10^8^	7.7840 × 10^7^	1.6920 × 10^6^	1.5027 × 10^6^
GSA	1.3287 × 10^3^	7.1522 × 10^1^	1.6371 × 10^6^	5.4089 × 10^5^	2.3191 × 10^5^	7.7187 × 10^4^
CPA	1.1680 × 10^3^	3.1242 × 10^1^	1.2730 × 10^6^	1.1778 × 10^6^	3.9934 × 10^3^	3.3746 × 10^3^
WFO	1.1800 × 10^3^	2.5985 × 10^1^	1.0070 × 10^6^	1.3891 × 10^6^	2.3452 × 10^4^	1.2798 × 10^4^
	**F13**		**F14**		**F15**	
	**Avg**	**Std**	**Avg**	**Std**	**Avg**	**Std**
SMGO	1.4310 × 10^3^	1.4149 × 10^1^	1.5179 × 10^3^	6.5685 × 10^0^	2.1782 × 10^3^	1.7438 × 10^2^
MGO	1.6733 × 10^4^	1.4209 × 10^4^	1.6875 × 10^4^	1.4668 × 10^4^	2.1974 × 10^3^	1.4184 × 10^2^
WOA	6.1462 × 10^5^	6.0835 × 10^5^	6.8159 × 10^4^	4.7416 × 10^4^	3.4429 × 10^3^	5.3256 × 10^2^
GWO	3.3077 × 10^5^	4.0944 × 10^5^	4.3115 × 10^5^	8.4938 × 10^5^	2.3434 × 10^3^	2.8100 × 10^2^
MFO	4.6599 × 10^5^	1.9112 × 10^6^	4.3333 × 10^4^	3.3102 × 10^4^	3.1616 × 10^3^	4.1797 × 10^2^
HGS	5.1178 × 10^4^	4.0034 × 10^4^	1.8666 × 10^4^	1.5543 × 10^4^	2.7629 × 10^3^	3.5257 × 10^2^
SCA	1.1012 × 10^5^	5.5586 × 10^4^	1.5510 × 10^7^	1.4539 × 10^7^	3.5903 × 10^3^	2.4133 × 10^2^
SMA	1.9436 × 10^5^	8.7743 × 10^4^	2.0506 × 10^4^	9.4838 × 10^3^	2.9293 × 10^3^	3.1443 × 10^2^
GSA	3.0862 × 10^4^	2.9272 × 10^4^	3.4449 × 10^4^	1.0824 × 10^4^	2.9379 × 10^3^	3.0179 × 10^2^
CPA	5.4338 × 10^3^	3.5405 × 10^3^	2.1990 × 10^3^	1.0398 × 10^3^	2.8333 × 10^3^	3.0258 × 10^2^
WFO	7.3156 × 10^3^	6.7038 × 10^3^	1.0309 × 10^4^	6.4121 × 10^3^	2.1477 × 10^3^	1.0709 × 10^2^
	**F16**		**F17**		**F18**	
	**Avg**	**Std**	**Avg**	**Std**	**Avg**	**Std**
SMGO	1.9002 × 10^3^	1.0447 × 10^2^	1.8397 × 10^3^	1.9859 × 10^1^	1.9158 × 10^3^	5.5399 × 10^0^
MGO	1.8600 × 10^3^	4.2152 × 10^1^	3.7571 × 10^5^	2.3069 × 10^5^	1.5664 × 10^4^	1.2781 × 10^4^
WOA	2.6091 × 10^3^	2.7531 × 10^2^	3.2724 × 10^6^	3.0579 × 10^6^	2.4860 × 10^6^	2.1769 × 10^6^
GWO	1.9861 × 10^3^	1.5501 × 10^2^	4.9097 × 10^5^	4.8152 × 10^5^	1.0569 × 10^6^	2.3215 × 10^6^
MFO	2.5034 × 10^3^	2.1322 × 10^2^	3.8499 × 10^6^	6.2796 × 10^6^	8.5973 × 10^6^	3.5575 × 10^7^
HGS	2.2226 × 10^3^	2.2916 × 10^2^	3.5957 × 10^5^	3.0060 × 10^5^	1.7356 × 10^4^	1.6978 × 10^4^
SCA	2.3935 × 10^3^	1.8099 × 10^2^	3.4647 × 10^6^	2.2299 × 10^6^	2.6848 × 10^7^	1.4593 × 10^7^
SMA	2.2460 × 10^3^	1.9883 × 10^2^	3.8694 × 10^5^	2.6001 × 10^5^	3.1965 × 10^5^	4.3610 × 10^5^
GSA	2.5412 × 10^3^	2.2601 × 10^2^	1.6154 × 10^5^	1.8744 × 10^5^	3.2916 × 10^4^	1.8404 × 10^4^
CPA	2.1268 × 10^3^	2.4248 × 10^2^	8.9246 × 10^4^	3.8600 × 10^4^	5.1004 × 10^3^	1.8305 × 10^3^
WFO	1.8362 × 10^3^	5.2909 × 10^1^	2.0200 × 10^5^	1.0310 × 10^5^	7.9903 × 10^3^	7.6128 × 10^3^
	**F19**		**F20**		**F21**	
	**Avg**	**Std**	**Avg**	**Std**	**Avg**	**Std**
SMGO	2.2258 × 10^3^	8.4839 × 10^1^	2.3654 × 10^3^	1.3001 × 10^1^	2.7372 × 10^3^	9.9730 × 10^2^
MGO	2.2424 × 10^3^	9.0168 × 10^1^	2.3720 × 10^3^	1.2837 × 10^1^	3.6254 × 10^3^	1.7748 × 10^3^
WOA	2.7199 × 10^3^	1.8330 × 10^2^	2.5721 × 10^3^	6.9775 × 10^1^	6.8975 × 10^3^	2.0153 × 10^3^
GWO	2.4290 × 10^3^	1.4299 × 10^2^	2.3788 × 10^3^	2.0536 × 10^1^	4.7636 × 10^3^	1.6194 × 10^3^
MFO	2.7163 × 10^3^	1.8093 × 10^2^	2.5070 × 10^3^	3.5413 × 10^1^	6.6496 × 10^3^	1.4332 × 10^3^
HGS	2.4964 × 10^3^	2.1179 × 10^2^	2.4194 × 10^3^	2.5884 × 10^1^	4.8492 × 10^3^	1.4857 × 10^3^
SCA	2.6254 × 10^3^	1.3317 × 10^2^	2.5545 × 10^3^	1.6906 × 10^1^	8.0356 × 10^3^	2.4955 × 10^3^
SMA	2.4653 × 10^3^	1.4729 × 10^2^	2.4682 × 10^3^	2.5278 × 10^1^	4.4861 × 10^3^	2.2753 × 10^3^
GSA	2.7994 × 10^3^	2.7742 × 10^2^	2.3613 × 10^3^	4.4359 × 10^1^	2.4126 × 10^3^	4.8371 × 10^2^
CPA	2.4532 × 10^3^	1.8445 × 10^2^	2.3685 × 10^3^	8.7339 × 10^1^	3.1456 × 10^3^	1.5765 × 10^3^
WFO	2.1818 × 10^3^	8.2755 × 10^1^	2.3539 × 10^3^	9.2548 × 10^0^	2.5125 × 10^3^	7.8849 × 10^2^
	**F22**		**F23**		**F24**	
	**Avg**	**Std**	**Avg**	**Std**	**Avg**	**Std**
SMGO	2.7046 × 10^3^	6.0293 × 10^1^	2.9321 × 10^3^	3.4263 × 10^1^	2.8859 × 10^3^	1.6256 × 10^0^
MGO	2.7192 × 10^3^	1.2345 × 10^1^	2.8958 × 10^3^	1.4767 × 10^1^	2.8875 × 10^3^	1.0732 × 10^0^
WOA	3.0472 × 10^3^	8.7824 × 10^1^	3.1510 × 10^3^	9.3738 × 10^1^	2.9446 × 10^3^	2.6137 × 10^1^
GWO	2.7475 × 10^3^	3.1981 × 10^1^	2.9086 × 10^3^	3.0960 × 10^1^	2.9855 × 10^3^	4.9034 × 10^1^
MFO	2.8392 × 10^3^	3.6469 × 10^1^	2.9940 × 10^3^	3.2593 × 10^1^	3.2080 × 10^3^	3.3724 × 10^2^
HGS	2.7732 × 10^3^	2.1987 × 10^1^	3.0253 × 10^3^	4.7815 × 10^1^	2.8863 × 10^3^	2.8191 × 10^0^
SCA	2.9981 × 10^3^	3.3245 × 10^1^	3.1577 × 10^3^	2.0521 × 10^1^	3.2073 × 10^3^	5.1133 × 10^1^
SMA	2.8669 × 10^3^	2.9060 × 10^1^	3.0227 × 10^3^	3.8477 × 10^1^	2.9941 × 10^3^	3.3764 × 10^1^
GSA	2.7066 × 10^3^	7.1872 × 10^1^	2.8319 × 10^3^	5.6729 × 10^1^	2.8910 × 10^3^	2.9788 × 10^0^
CPA	2.7536 × 10^3^	2.7725 × 10^1^	3.0528 × 10^3^	7.3253 × 10^1^	2.8958 × 10^3^	1.5871 × 10^1^
WFO	2.7076 × 10^3^	1.3687 × 10^1^	2.8786 × 10^3^	5.0620 × 10^1^	2.8868 × 10^3^	9.0176 × 10^−1^
	**F25**		**F26**		**F27**	
	**Avg**	**Std**	**Avg**	**Std**	**Avg**	**Std**
SMGO	3.8616 × 10^3^	7.9115 × 10^2^	3.2141 × 10^3^	1.2701 × 10^1^	3.1321 × 10^3^	4.9880 × 10^1^
MGO	4.0189 × 10^3^	4.7639 × 10^2^	3.2137 × 10^3^	5.0916 × 10^0^	3.2301 × 10^3^	1.7783 × 10^1^
WOA	7.5711 × 10^3^	1.1560 × 10^3^	3.3603 × 10^3^	7.1863 × 10^1^	3.3075 × 10^3^	2.6743 × 10^1^
GWO	4.6268 × 10^3^	3.6738 × 10^2^	3.2526 × 10^3^	1.9282 × 10^1^	3.4217 × 10^3^	1.2640 × 10^2^
MFO	5.9357 × 10^3^	4.6263 × 10^2^	3.2567 × 10^3^	2.6800 × 10^1^	4.4730 × 10^3^	1.0222 × 10^3^
HGS	5.0841 × 10^3^	3.4906 × 10^2^	3.2249 × 10^3^	1.3380 × 10^1^	3.1932 × 10^3^	5.0991 × 10^1^
SCA	6.9029 × 10^3^	3.1439 × 10^2^	3.4032 × 10^3^	4.2956 × 10^1^	3.8524 × 10^3^	1.5756 × 10^2^
SMA	5.2923 × 10^3^	6.5437 × 10^2^	3.2550 × 10^3^	2.7206 × 10^1^	3.4027 × 10^3^	5.2622 × 10^1^
GSA	3.0055 × 10^3^	3.6318 × 10^2^	3.2240 × 10^3^	1.7086 × 10^1^	3.1255 × 10^3^	3.5247 × 10^1^
CPA	4.3341 × 10^3^	1.2806 × 10^3^	3.2365 × 10^3^	1.7104 × 10^1^	3.1779 × 10^3^	5.8120 × 10^1^
WFO	3.9509 × 10^3^	4.5044 × 10^2^	3.2092 × 10^3^	6.4143 × 10^0^	3.2235 × 10^3^	1.4031 × 10^1^
	**F28**		**F29**			
	**Avg**	**Std**	**Avg**	**Std**		
SMGO	3.4571 × 10^3^	9.1496 × 10^1^	5.1794 × 10^3^	2.5748 × 10^2^		
MGO	3.6075 × 10^3^	7.4577 × 10^1^	9.1958 × 10^4^	8.5328 × 10^4^		
WOA	4.7991 × 10^3^	4.6821 × 10^2^	1.2005 × 10^7^	7.3847 × 10^6^		
GWO	3.7619 × 10^3^	1.5634 × 10^2^	6.2539 × 10^6^	8.8408 × 10^6^		
MFO	4.1786 × 10^3^	2.6042 × 10^2^	1.5915 × 10^6^	3.0360 × 10^6^		
HGS	3.7251 × 10^3^	1.7567 × 10^2^	1.4972 × 10^5^	1.8399 × 10^5^		
SCA	4.6096 × 10^3^	2.2003 × 10^2^	6.8042 × 10^7^	1.9596 × 10^7^		
SMA	4.0310 × 10^3^	2.1010 × 10^2^	5.8803 × 10^6^	3.5710 × 10^6^		
GSA	4.0442 × 10^3^	1.9451 × 10^2^	1.7127 × 10^5^	8.6436 × 10^4^		
CPA	3.6885 × 10^3^	2.1159 × 10^2^	1.2161 × 10^4^	4.7346 × 10^3^		
WFO	3.5981 × 10^3^	5.4032 × 10^1^	5.4166 × 10^4^	2.9243 × 10^4^		
	**Overall Rank**					
	**RANK**	**+/=/−**	**AVG**			
SMGO	1	~	1.8966			
MGO	4	19/7/3	4.1034			
WOA	10	29/0/0	9.5172			
GWO	7	28/0/1	6.6207			
MFO	9	29/0/0	9.3103			
HGS	6	28/1/0	5.4138			
SCA	11	29/0/0	9.8966			
SMA	8	29/0/0	7.8621			
GSA	5	21/4/4	4.8621			
CPA	3	25/2/2	4.069			
WFO	2	15/5/9	2.4483			

**Table 2:** The *p*-values of the SMGO versus other algorithms on CEC2017.

	WFO	MGO	WOA	GWO	MFO
F1	1.734 × 10^−6^	1.734 × 10^−6^	1.734 × 10^−6^	1.734 × 10^−6^	1.733 × 10^−6^
F2	1.734 × 10^−6^	1.734 × 10^−6^	1.734 × 10^−6^	1.734 × 10^−6^	1.734 × 10^−6^
F3	1.734 × 10^−6^	1.734 × 10^−6^	1.734 × 10^−6^	1.734 × 10^−6^	1.734 × 10^−6^
F4	2.849 × 10^−2^	3.185 × 10^−1^	1.734 × 10^−6^	1.734 × 10^−6^	1.734 × 10^−6^
F5	4.449 × 10^−5^	2.067 × 10^−2^	1.734 × 10^−6^	1.734 × 10^−6^	1.734 × 10^−6^
F6	1.480 × 10^−2^	8.217 × 10^−3^	1.734 × 10^−6^	2.603 × 10^−6^	1.734 × 10^−6^
F7	2.105 × 10^−3^	3.872 × 10^−2^	1.734 × 10^−6^	1.494 × 10^−5^	1.734 × 10^−6^
F8	1.921 × 10^−6^	3.001 × 10^−2^	1.734 × 10^−6^	1.921 × 10^−6^	1.734 × 10^−6^
F9	3.182 × 10^−6^	1.734 × 10^−6^	1.734 × 10^−6^	1.360 × 10^−5^	1.734 × 10^−6^
F10	1.734 × 10^−6^	1.734 × 10^−6^	1.734 × 10^−6^	1.734 × 10^−6^	1.734 × 10^−6^
F11	1.734 × 10^−6^	1.734 × 10^−6^	1.734 × 10^−6^	1.734 × 10^−6^	1.734 × 10^−6^
F12	1.734 × 10^−6^	1.734 × 10^−6^	1.734 × 10^−6^	1.734 × 10^−6^	1.734 × 10^−6^
F13	1.734 × 10^−6^	1.734 × 10^−6^	1.734 × 10^−6^	1.734 × 10^−6^	1.734 × 10^−6^
F14	1.734 × 10^−6^	1.734 × 10^−6^	1.734 × 10^−6^	1.734 × 10^−6^	1.734 × 10^−6^
F15	3.600 × 10^−1^	6.583 × 10^−1^	1.734 × 10^−6^	2.431 × 10^−2^	1.734 × 10^−6^
F16	1.752 × 10^−2^	1.306 × 10^−1^	2.127 × 10^−6^	3.854 × 10^−3^	1.734 × 10^−6^
F17	1.734 × 10^−6^	1.734 × 10^−6^	1.734 × 10^−6^	1.734 × 10^−6^	1.734 × 10^−6^
F18	1.734 × 10^−6^	1.734 × 10^−6^	1.734 × 10^−6^	1.734 × 10^−6^	1.734 × 10^−6^
F19	9.368 × 10^−2^	3.820 × 10^−1^	1.734 × 10^−6^	1.360 × 10^−5^	1.921 × 10^−6^
F20	8.307 × 10^−4^	1.752 × 10^−2^	1.734 × 10^−6^	9.842 × 10^−3^	1.734 × 10^−6^
F21	3.683 × 10^−2^	9.711 × 10^−5^	1.734 × 10^−6^	1.360 × 10^−5^	3.182 × 10^−6^
F22	2.289 × 10^−1^	2.452 × 10^−1^	1.734 × 10^−6^	1.605 × 10^−4^	1.734 × 10^−6^
F23	5.752 × 10^−6^	9.711 × 10^−5^	1.734 × 10^−6^	5.667 × 10^−3^	8.466 × 10^−6^
F24	9.842 × 10^−3^	3.515 × 10^−6^	1.734 × 10^−6^	1.734 × 10^−6^	1.734 × 10^−6^
F25	7.499 × 10^−1^	4.165 × 10^−1^	1.734 × 10^−6^	1.605 × 10^−4^	1.734 × 10^−6^
F26	5.984 × 10^−2^	5.999 × 10^−1^	1.734 × 10^−6^	2.127 × 10^−6^	1.734 × 10^−6^
F27	2.127 × 10^−6^	1.734 × 10^−6^	1.734 × 10^−6^	1.734 × 10^−6^	1.734 × 10^−6^
F28	2.843 × 10^−5^	6.320 × 10^−5^	1.734 × 10^−6^	2.353 × 10^−6^	1.734 × 10^−6^
F29	1.734 × 10^−6^	1.734 × 10^−6^	1.734 × 10^−6^	1.734 × 10^−6^	1.734 × 10^−6^
	**HGS**	**SCA**	**SMA**	**GSA**	**CPA**
F1	1.734 × 10^−6^	1.734 × 10^−6^	1.734 × 10^−6^	1.734 × 10^−6^	1.734 × 10^−6^
F2	1.734 × 10^−6^	1.734 × 10^−6^	1.734 × 10^−6^	1.734 × 10^−6^	1.734 × 10^−6^
F3	1.734 × 10^−6^	1.734 × 10^−6^	1.734 × 10^−6^	2.370 × 10^−5^	7.691 × 10^−6^
F4	1.734 × 10^−6^	1.734 × 10^−6^	1.734 × 10^−6^	2.585 × 10^−3^	2.127 × 10^−6^
F5	1.734 × 10^−6^	1.734 × 10^−6^	1.734 × 10^−6^	1.734 × 10^−6^	2.843 × 10^−5^
F6	1.734 × 10^−6^	1.734 × 10^−6^	1.734 × 10^−6^	1.734 × 10^−6^	3.182 × 10^−6^
F7	2.353 × 10^−6^	1.734 × 10^−6^	1.734 × 10^−6^	1.589 × 10^−1^	1.734 × 10^−6^
F8	1.734 × 10^−6^	1.734 × 10^−6^	1.734 × 10^−6^	1.734 × 10^−6^	2.353 × 10^−6^
F9	5.216 × 10^−6^	1.734 × 10^−6^	1.734 × 10^−6^	2.765 × 10^−3^	1.921 × 10^−6^
F10	1.734 × 10^−6^	1.734 × 10^−6^	1.734 × 10^−6^	1.734 × 10^−6^	6.339 × 10^−6^
F11	1.734 × 10^−6^	1.734 × 10^−6^	1.734 × 10^−6^	1.734 × 10^−6^	1.734 × 10^−6^
F12	1.734 × 10^−6^	1.734 × 10^−6^	1.734 × 10^−6^	1.734 × 10^−6^	1.921 × 10^−6^
F13	1.734 × 10^−6^	1.734 × 10^−6^	1.734 × 10^−6^	1.734 × 10^−6^	1.734 × 10^−6^
F14	1.734 × 10^−6^	1.734 × 10^−6^	1.734 × 10^−6^	1.734 × 10^−6^	1.734 × 10^−6^
F15	3.515 × 10^−6^	1.734 × 10^−6^	1.921 × 10^−6^	1.734 × 10^−6^	2.879 × 10^−6^
F16	8.466 × 10^−6^	1.734 × 10^−6^	2.127 × 10^−6^	1.734 × 10^−6^	2.412 × 10^−4^
F17	1.734 × 10^−6^	1.734 × 10^−6^	1.734 × 10^−6^	1.734 × 10^−6^	1.734 × 10^−6^
F18	1.734 × 10^−6^	1.734 × 10^−6^	1.734 × 10^−6^	1.734 × 10^−6^	1.734 × 10^−6^
F19	7.691 × 10^−6^	1.734 × 10^−6^	4.729 × 10^−6^	1.921 × 10^−6^	2.370 × 10^−5^
F20	2.603 × 10^−6^	1.734 × 10^−6^	1.734 × 10^−6^	1.359 × 10^−1^	1.650 × 10^−1^
F21	2.597 × 10^−5^	2.603 × 10^−6^	3.065 × 10^−4^	4.950 × 10^−2^	3.308 × 10^−3^
F22	1.734 × 10^−6^	1.734 × 10^−6^	1.734 × 10^−6^	7.036 × 10^−1^	2.353 × 10^−6^
F23	2.603 × 10^−6^	1.734 × 10^−6^	1.734 × 10^−6^	1.734 × 10^−6^	5.216 × 10^−6^
F24	3.709 × 10^−1^	1.734 × 10^−6^	1.734 × 10^−6^	2.603 × 10^−6^	4.897 × 10^−4^
F25	2.353 × 10^−6^	1.734 × 10^−6^	5.752 × 10^−6^	1.197 × 10^−3^	9.363 × 10^−2^
F26	1.175 × 10^−2^	1.734 × 10^−6^	3.882 × 10^−6^	7.731 × 10^−3^	2.370 × 10^−5^
F27	1.057 × 10^−4^	1.734 × 10^−6^	1.734 × 10^−6^	6.288 × 10^−1^	7.731 × 10^−3^
F28	1.494 × 10^−5^	1.734 × 10^−6^	1.734 × 10^−6^	1.734 × 10^−6^	6.892 × 10^−5^
F29	1.734 × 10^−6^	1.734 × 10^−6^	1.734 × 10^−6^	1.734 × 10^−6^	1.734 × 10^−6^

**Table 3:** Results of the SMGO and Other Algorithms on Classic Benchmark Test Functions.

	Sphere		Schwefel 2.22		Rosenbrock	
	**Avg**	**Std**	**Avg**	**Std**	**Avg**	**Std**
SMGO	0.000 × 10^0^	0.000 × 10^0^	0.000 × 10^0^	0.000 × 10^0^	6.131 × 10^−16^	3.358 × 10^−15^
MGO	2.373 × 10^−7^	1.289 × 10^−6^	2.594 × 10^−14^	1.421 × 10^−13^	1.741 × 10^1^	1.737 × 10^1^
WOA	0.000 × 10^0^	0.000 × 10^0^	0.000 × 10^0^	0.000 × 10^0^	1.455 × 10^1^	2.550 × 10^−1^
GWO	0.000 × 10^0^	0.000 × 10^0^	2.261 × 10^−298^	0.000 × 10^0^	1.636 × 10^1^	7.009 × 10^−1^
MFO	4.194 × 10^−92^	2.233 × 10^−91^	1.467 × 10^1^	1.167 × 10^1^	1.255 × 10^4^	3.092 × 10^4^
HGS	0.000 × 10^0^	0.000 × 10^0^	0.000 × 10^0^	0.000 × 10^0^	4.323 × 10^0^	2.736 × 10^0^
SCA	1.925 × 10^−64^	1.051 × 10^−63^	1.311 × 10^−60^	6.426 × 10^−60^	1.703 × 10^1^	5.165 × 10^−1^
SMA	0.000 × 10^0^	0.000 × 10^0^	0.000 × 10^0^	0.000 × 10^0^	4.507 × 10^−2^	8.108 × 10^−2^
GSA	8.725 × 10^0^	1.229 × 10^0^	1.119 × 10^1^	7.649 × 10^−1^	3.346 × 10^3^	8.216 × 10^2^
CPA	0.000 × 10^0^	0.000 × 10^0^	0.000 × 10^0^	0.000 × 10^0^	1.306 × 10^1^	1.501 × 10^−1^
WFO	1.114 × 10^−6^	6.099 × 10^−6^	3.906 × 10^−16^	1.389 × 10^−15^	1.788 × 10^1^	1.674 × 10^1^
	**Rastrigin**		**Ackley**		**Griewank**	
	**Avg**	**Std**	**Avg**	**Std**	**Avg**	**Std**
SMGO	0.000 × 10^0^	0.000 × 10^0^	9.652 × 10^−15^	3.057 × 10^−15^	7.392 × 10^−4^	2.976 × 10^−3^
MGO	3.783 × 10^0^	1.679 × 10^0^	7.271 × 10^−5^	3.502 × 10^−4^	3.610 × 10^−3^	7.904 × 10^−3^
WOA	0.000 × 10^0^	0.000 × 10^0^	3.730 × 10^−15^	1.957 × 10^−15^	1.617 × 10^−3^	6.822 × 10^−3^
GWO	0.000 × 10^0^	0.000 × 10^0^	5.151 × 10^−15^	1.445 × 10^−15^	5.793 × 10^−4^	2.229 × 10^−3^
MFO	7.878 × 10^1^	2.908 × 10^1^	4.390 × 10^0^	7.837 × 10^0^	6.087 × 10^0^	2.305 × 10^1^
HGS	0.000 × 10^0^	0.000 × 10^0^	8.882 × 10^−16^	0.000 × 10^0^	0.000 × 10^0^	0.000 × 10^0^
SCA	4.575 × 10^−4^	2.506 × 10^−3^	3.001 × 10^0^	6.515 × 10^0^	6.592 × 10^−3^	2.584 × 10^−2^
SMA	0.000 × 10^0^	0.000 × 10^0^	8.882 × 10^−16^	0.000 × 10^0^	0.000 × 10^0^	0.000 × 10^0^
GSA	1.221 × 10^2^	7.181 × 10^0^	3.991 × 10^0^	2.081 × 10^−1^	5.139 × 10^−1^	5.320 × 10^−2^
CPA	0.000 × 10^0^	0.000 × 10^0^	8.882 × 10^−16^	0.000 × 10^0^	0.000 × 10^0^	0.000 × 10^0^
WFO	4.645 × 10^0^	1.907 × 10^0^	6.812 × 10^−5^	2.682 × 10^−4^	1.918 × 10^−3^	7.216 × 10^−3^
	**Overall Rank**					
	**RANK**	**+/=/−**				
SMGO	4	~				
MGO	8	6/0/0				
WOA	5	1/4/1				
GWO	6	2/3/1				
MFO	10	5/1/0				
HGS	2	1/4/1				
SCA	7	4/2/0				
SMA	1	1/4/1				
GSA	11	6/0/0				
CPA	3	1/4/1				
WFO	8	6/0/0				

**Table 4:** The results of SMGO and other algorithms on the oil reservoir production optimization.

Algorithm	NPV (USD)			
	Mean	Std	Best	Worst
SMGO	**1.0528 × 108**	**1.1534 × 106**	**1.0792 × 108**	**1.0251 × 108**
MGO	9.7145 × 107	2.1881 × 106	1.0133 × 108	9.2764 × 107
WFO	1.0039 × 107	1.8950 × 106	1.0458 × 108	9.6822 × 107
CPA	9.8821 × 107	2.0547 × 106	1.0296 × 108	9.4539 × 107
GSA	9.6503 × 107	2.3166 × 106	1.0075 × 108	9.1880 × 107
HGS	9.4972 × 107	2.4019 × 106	9.9824 × 108	9.0157 × 107
GWO	9.3160 × 107	2.6785 × 106	9.8051 × 107	8.8305 × 107
SMA	9.0884 × 107	2.9523 × 106	9.6177 × 107	8.5492 × 107
WOA	8.9526 × 107	3.1048 × 106	9.4988 × 107	8.4116 × 107
MFO	8.7739 × 107	3.3512 × 106	9.2843 × 107	8.1963 × 107
SCA	8.5015 × 107	3.7891 × 106	9.1025 × 107	7.9348 × 107

## Data Availability

The numerical and experimental data used to support the findings of this study are included within the article.

## References

[1] Faris H., Al-Zoubi A.M., Heidari A.A., Aljarah I., Mafarja M., Hassonah M.A., Fujita H. (2019). An Intelligent System for Spam Detection and Identification of the Most Relevant Features Based on Evolutionary Random Weight Networks. Inf. Fusion.

[2] Chong E.K., Zak S.H. (2004). An Introduction to Optimization.

[3] Ren X., Wang H., Hu H., Wang J., Ablameyko S.V. (2025). Weighted Committee-Based Surrogate-Assisted Differential Evolution Framework for Efficient Medium-Scale Expensive Optimization. Int. J. Mach. Learn. Cybern..

[4] Song G., Song X., Li G., Shi Y., Wang G., Ji J., Xu F., Song Z. (2021). An Integrated Multi-Objective Optimization Method to Improve the Performance of Multilateral-Well Geothermal System. Renew. Energy.

[5] Clarke J., McLay L., McLeskey J.T. (2014). Comparison of Genetic Algorithm to Particle Swarm for Constrained Simulation-Based Optimization of a Geothermal Power Plant. Adv. Eng. Inform..

[6] Garg T., Kaur G., Rana P.S., Cheng X. (2024). Enhancing Road Traffic Prediction Using Data Preprocessing Optimization. J. Circuits Syst. Comput..

[7] Wang Z., Wu L., Li B., Cheng Y., Li X., Wang X., Han L., Wu X., Fan Y., Yu Y. (2023). Toripalimab plus Chemotherapy for Patients with Treatment-Naive Advanced Non-Small-Cell Lung Cancer: A Multicenter Randomized Phase III Trial (CHOIC × 10^−1^). J. Clin. Oncol. Off. J. Am. Soc. Clin. Oncol..

[8] Sitinjak C., Johanna A., Avinash B., Bevoor B. (2023). Financial Management: A System of Relations for Optimizing Enterprise Finances—A Review. J. Markcount Financ..

[9] Huang X., Hu H., Wang J., Yuan B., Dai C., Ablameyk S.V. Dynamic Strongly Convex Sparse Operator with Learning Mechanism for Sparse Large-Scale Multi-Objective Optimization. Proceedings of the 2024 6th International Conference on Data-driven Optimization of Complex Systems (DOCS).

[10] Hu H., Wang J., Huang X., Ablameyko S.V. An Integrated Online-Offline Hybrid Particle Swarm Optimization Framework for Medium Scale Expensive Problems*. Proceedings of the 2024 6th International Conference on Data-driven Optimization of Complex Systems (DOCS).

[11] Luu M.T., Manaf N.A., Abbas A. (2015). Dynamic Modelling and Control Strategies for Flexible Operation of Amine-Based Post-Combustion CO_2_ Capture Systems. Int. J. Greenh. GAS Control..

[12] Varelas K., Auger A., Brockhoff D., Hansen N., ElHara O.A., Semet Y., Kassab R., Barbaresco F., Auger A., Fonseca C.M., Lourenço N., Machado P., Paquete L., Whitley D. (2018). A Comparative Study of Large-Scale Variants of CMA-ES. Parallel Problem Solving from Nature—PPSN XV.

[13] Polyak B.T. (1969). The Conjugate Gradient Method in Extremal Problems. USSR Comput. Math. Math. Phys..

[14] Potra F.A., Wright S.J. (2000). Interior-Point Methods. J. Comput. Appl. Math..

[15] Bottou L., Curtis F.E., Nocedal J. (2018). Optimization Methods for Large-Scale Machine Learning. SIAM Rev..

[16] Hu H., Shan W., Tang Y., Heidari A.A., Chen H., Liu H., Wang M., Escorcia-Gutierrez J., Mansour R.F., Chen J. (2022). Horizontal and Vertical Crossover of Sine Cosine Algorithm with Quick Moves for Optimization and Feature Selection. J. Comput. Des. Eng..

[17] Shan W., Hu H., Cai Z., Chen H., Liu H., Wang M., Teng Y. (2022). Multi-Strategies Boosted Mutative Crow Search Algorithm for Global Tasks: Cases of Continuous and Discrete Optimization. J. Bionic Eng..

[18] Hu H., Shan W., Chen J., Xing L., Heidari A.A., Chen H., He X., Wang M. (2023). Dynamic Individual Selection and Crossover Boosted Forensic-Based Investigation Algorithm for Global Optimization and Feature Selection. J. Bionic Eng..

[19] Akay B., Karaboga D. (2012). Artificial Bee Colony Algorithm for Large-Scale Problems and Engineering Design Optimization. J. Intell. Manuf..

[20] Yu W.-J., Shen M., Chen W.-N., Zhan Z.-H., Gong Y.-J., Lin Y., Liu O., Zhang J. (2013). Differential Evolution with Two-Level Parameter Adaptation. IEEE Trans. Cybern..

[21] Chou J.-S., Nguyen N.-M. (2020). FBI Inspired Meta-Optimization. Appl. Soft Comput..

[22] Dokeroglu T., Deniz A., Kiziloz H.E. (2022). A Comprehensive Survey on Recent Metaheuristics for Feature Selection. Neurocomputing.

[23] Das S., Suganthan P.N. (2010). Differential Evolution: A Survey of the State-of-the-Art. IEEE Trans. Evol. Comput..

[24] Bertsimas D., Tsitsiklis J. (1993). Simulated Annealing. Statist. Sci..

[25] Rashedi E., Nezamabadi-Pour H., Saryazdi S. (2009). GSA: A Gravitational Search Algorithm. Inf. Sci..

[26] Poli R., Kennedy J., Blackwell T. (2007). Particle Swarm Optimization. Swarm Intell..

[27] Dorigo M., Birattari M., Stutzle T. (2006). Ant Colony Optimization. IEEE Comput. Intell. Mag..

[28] Zheng B., Chen Y., Wang C., Heidari A.A., Liu L., Chen H. (2024). The Moss Growth Optimization (MGO): Concepts and Performance. J. Comput. Des. Eng..

[29] Ho Y.C., Pepyne D.L. (2002). Simple Explanation of the No-Free-Lunch Theorem and Its Implications. J. Optim. Theory Appl..

[30] Wolpert D.H., Macready W.G. (1997). No Free Lunch Theorems for Optimization. IEEE Trans. Evol. Comput..

[31] Wang L., Yao Y., Zhao G., Adenutsi C.D., Wang W., Lai F. (2022). A Hybrid Surrogate-Assisted Integrated Optimization of Horizontal Well Spacing and Hydraulic Fracture Stage Placement in Naturally Fractured Shale Gas Reservoir. J. Petrol. Sci. Eng..

[32] Yin F., Xue X., Zhang C., Zhang K., Han J., Liu B., Wang J., Yao J. (2021). Multifidelity Genetic Transfer: An Efficient Framework for Production Optimization. SPE J..

[33] Dai Q., Zhang L., Zhang K., Chen G., Ma X., Wang J., Zhang H., Yan X., Liu P., Yang Y. (2023). An Efficient Infill Well Placement Optimization Approach for Extra-Low Permeability Reservoir. J. Energy Resour. Technol.-Trans. ASME.

[34] Bertini J.R., Ferreira Batista S., Funcia M.A., Mendes Da Silva L.O., Santos A.A.S., Schiozer D.J. (2022). A Comparison of Machine Learning Surrogate Models for Net Present Value Prediction from Well Placement Binary Data. J. Petrol. Sci. Eng..

[35] Duplyakov V.M., Morozov A.D., Popkov D.O., Shel E.V., Vainshtein A.L., Burnaev E.V., Osiptsov A.A., Paderin G.V. (2022). Data-Driven Model for Hydraulic Fracturing Design Optimization. Part II: Inverse Problem. J. Petrol. Sci. Eng..

[36] Kasravi J., Safarzadeh M.A., Hashemi A. (2017). A Population-Feedback Control Based Algorithm for Well Trajectory Optimization Using Proxy Model. J. Rock Mech. Geotech. Eng..

[37] Chen G., Li Y., Zhang K., Xue X., Wang J., Luo Q., Yao C., Yao J. (2021). Efficient Hierarchical Surrogate-Assisted Differential Evolution for High-Dimensional Expensive Optimization. Inf. Sci..

[38] Gao Y., Cheng L. (2025). A Multi-Swarm Greedy Selection Enhanced Fruit Fly Optimization Algorithm for Global Optimization in Oil and Gas Production. PLoS ONE.

[39] Yue T., Li T. (2025). Crisscross Moss Growth Optimization: An Enhanced Bio-Inspired Algorithm for Global Production and Optimization. Biomimetics.

[40] Wu G., Mallipeddi R., Suganthan P.N. (2017). Problem Definitions and Evaluation Criteria for the CEC 2017 Competition on Constrained Real-Parameter Optimization.

[41] Mirjalili S., Lewis A. (2016). The Whale Optimization Algorithm. Adv. Eng. Softw..

[42] Mirjalili S., Mirjalili S.M., Lewis A. (2014). Grey Wolf Optimizer. Adv. Eng. Softw..

[43] Mirjalili S. (2015). Moth-Flame Optimization Algorithm: A Novel Nature-Inspired Heuristic Paradigm. Knowl.-Based Syst..

[44] Yang Y., Chen H., Heidari A.A., Gandomi A.H. (2021). Hunger Games Search: Visions, Conception, Implementation, Deep Analysis, Perspectives, and towards Performance Shifts. Expert Syst. Appl..

[45] Mirjalili S. (2016). SCA: A Sine Cosine Algorithm for Solving Optimization Problems. Knowl.-Based Syst..

[46] Li S., Chen H., Wang M., Heidari A.A., Mirjalili S. (2020). Slime Mould Algorithm: A New Method for Stochastic Optimization. Future Gener. Comput. Syst..

[47] Tu J., Chen H., Wang M., Gandomi A.H. (2021). The Colony Predation Algorithm. J. Bionic Eng..

[48] Luo K. (2022). Water Flow Optimizer: A Nature-Inspired Evolutionary Algorithm for Global Optimization. IEEE Trans. Cybern..

